# Self-Assessed Competency at Working with a Medical Interpreter Is Not Associated with Knowledge of Good Practice

**DOI:** 10.1371/journal.pone.0038973

**Published:** 2012-06-08

**Authors:** Patricia Hudelson, Thomas Perneger, Véronique Kolly, Noëlle Junod Perron

**Affiliations:** 1 Department of Community Medicine, Primary Care and Emergency Medicine, University Hospitals of Geneva, Geneva, Switzerland; 2 Department of Community Health and Medicine, Faculty of Medicine, University of Geneva, Geneva, Switzerland; 3 Coordinator of Patient Relations, Espace Médiation, University Hospitals of Geneva, Geneva, Switzerland; RAND Corporation, United States of America

## Abstract

**Background:**

Specific knowledge and skills are needed to work effectively with an interpreter, but most doctors have received limited training. Self-assessed competency may not accurately identify training needs.

**Purposes:**

The purpose of this study is to explore the association between self-assessed competency at working with an interpreter and the ability to identify elements of good practice, using a written vignette.

**Methods:**

A mailed questionnaire was sent to 619 doctors and medical students in Geneva, Switzerland.

**Results:**

58.6% of respondents considered themselves to be highly competent at working with a professional interpreter, but 22% failed to mention even one element of good practice in response to the vignette, and only 39% could name more than one. There was no association between self-rated competency and number of elements mentioned.

**Conclusions:**

Training efforts should challenge the assumption that working with an interpreter is intuitive. Evaluation of clinicians' ability to work with an interpreter should not be limited to self-ratings. In the context of large-scale surveys, written vignettes may provide a simple method for identifying knowledge of good practice and topics requiring further training.

## Introduction

Language barriers between patients and health care providers are common, and are associated with poorer quality of care and lower patient satisfaction [1], [2], [3], [4]. Language assistance provided by trained, professional interpreters has been shown to improve health care utilization, clinical outcomes and satisfaction [1], [5], [6]. Many health care organizations now recommend that physicians work with professional interpreters in order to ensure patient safety, quality of care, appropriate patient participation in health care decisions, and informed consent [7], [8], [9].

Specific skills are needed to work effectively with an interpreter, and a large number of guidelines and training programs have been developed to foster effective collaboration between health care providers and medical interpreters [10], [11], [12],[13],[14],[15],[16],[17]. While some aspects of recommended practice may vary–for example some recommend positioning the interpreter next to but slightly behind the patient while others recommend that patient, doctor and translator sit in a triangle–most guidelines recommend some combination of the following: the doctor should conduct a pre-session discussion with the interpreter to clarify objectives and needs, introduce the interpreter to the patient and explain that he/she is held by a strict code of ethics to maintain confidentiality, ask the patient if he/she accepts the interpreter, speak directly to the patient, speak in short sentences and avoid medical jargon, and conduct a post-session discussion with the interpreter.

Despite clear guidelines regarding effective interpreter-mediated clinical communication, most doctors have received only limited training in how to work with an interpreter. While a few studies have looked at medical students' and residents' self-rated preparedness to work with interpreters [18], [19], the association between self-assessment and knowledge of how to work with an interpreter is unknown.

In order to examine these issues, we explored the association between doctors' and medical students' previously reported self-assessed competency at working with an interpreter [20] and their ability to name elements of good practice regarding working with an interpreter, as measured using a vignette (previously unpublished data).

## Methods

### Ethics statement

The study was approved by the research ethics committee at the University Hospitals of Geneva.

### Study population and data collection

Participants were doctors and medical students in Geneva, Switzerland. We selected a random sample of 600 physicians from a total of about 1400 physicians working in 11 medical departments at the University Hospitals of Geneva, a random sample of 600 physicians working in private practice in Geneva from a list of about 1800 physicians provided by the Geneva Medical Association, and all 250 local medical students in their clinical years (years 4, 5 and 6). The sample size was determined to provide enough power for the main objective of the study which was the analysis of physicians' attitudes and opinions regarding care of immigrant patients. [21] The analysis of interpreter-related knowledge was a secondary objective.

A self-administered questionnaire was mailed to respondents, and then sent again to non-respondents 4 and 8 weeks after the initial mailing. The first page of the questionnaire explained the objectives of the study, the voluntary nature of their participation, how anonymity of participants and the confidentiality of their responses would be protected, and the potential risks/benefits of their participation. Physicians were asked to check the appropriate box indicating whether or not he/she agreed to participate in the study, and to return the questionnaire (filled in or not, according to whether he/she has agreed to participate or not). We chose this approach because it allowed for consent to be explicit but not nominative.

### Questionnaire

The questionnaire [21] contained questions about respondents' sociodemographic and professional characteristics, as well as their attitudes, opinions and experiences regarding language barriers and interpreter-use:

- In order to explore the relative importance of language barriers for respondents, we developed a list of 15 potential sources of difficulty when caring for immigrant patients, and asked respondents to indicate whether each of the items was a rare or frequent cause of difficulty in their own work (scale of 1–5, 1 = rare, 5 = very frequent), as well as to indicate which they considered to be among the top 5 causes of difficulty (without ranking them). Among the list of 15 items, we included “insufficient knowledge of the local language (French) by the immigrant patient” and “use of friends or family members as interpreters” (Table).
- An open-ended question asked: “What categories of patients do you have the most difficulty communicating with or understanding?” The answers were grouped into categories by the investigators.
- The respondents rated their level of interest in taking care of immigrant patients, on a 5-point scale (from “none” to “very high”).
- Respondents' rated their competency at 14 clinical tasks (1 = not at all competent; 5 = perfectly competent). Self-rated competency results have been reported elsewhere [20]. One of the items asked respondents “How competent do you consider yourself at working with a professional interpreter?”

**Figure 1:**
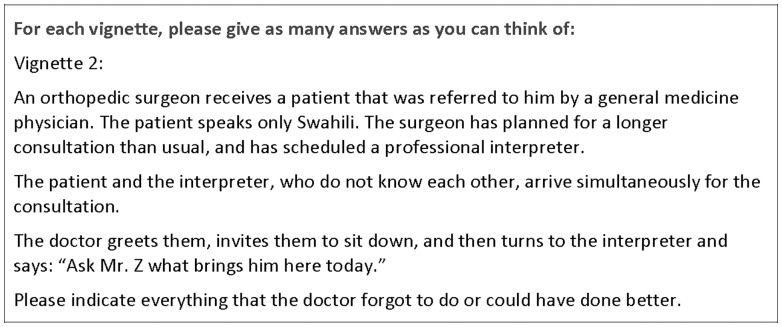
The interpreting vignette.

To explore respondents' knowledge of how to work with a professional interpreter, they were asked to read a brief vignette involving the first few minutes of an interpreted consultation, and to indicate (using brief, open-ended answers) anything the physician omitted to do or could have done better. They were encouraged to provide as many answers as possible (Figure 1). Typical answers provided by respondents included: “introduce interpreter to patient,” “speak directly to patient”, “explain confidentiality”.

The vignette was developed by the authors to reflect a common clinical situation. Drafts of the vignette were pre-tested with several clinicians not involved in the study to check for relevance and clarity.

### Statistical analysis

We examined the frequency distributions of all closed-format items (respondent characteristics, importance of difficulties, status as one of top five difficulties, self-assessed competence in working with interpreter).

For the vignette, we gave one point for each answer that reflected effective collaboration with an interpreter, based on published guidelines [10], [11], [14] and what is taught in continuing education seminars at our hospital [16]. This could include any of the following practices: scheduling sufficient time for the 3-way consultation; conducting a brief pre-session discussion with the interpreter to clarify your needs/expectations; encouraging the interpreter to indicate any problems he/she perceives; greeting the patient; explaining the interpreter's role; explaining interpreter confidentiality to the patient; asking if the patient accepts the interpreter; speaking directly to the patient; looking at the patient when he/she is speaking; avoiding jargon; speaking in short sentences and asking one question at a time; checking for patient's understanding; keeping control of the interview; conducting a post-session discussion with the interpreter.

Given the nature of the vignette (which only presents the beginning of the consultation), we anticipated that certain answers would be more likely, such as greeting the patient, introducing the interpreter to the patient, explaining confidentiality, verifying that the patient accepts the interpreter's presence, and speaking directly to the patient. However, we were not looking for any pre-determined number or set of answers, and points were assigned to any answers that reflected good practice.

The coding process consisted of all authors first reading through answers to the vignette and creating a consensus list of codes based on the content of respondents' answers. This resulted in a total of 5 codes: 1) the physician should introduce the interpreter to the patient; 2) the physician should mention interpreter confidentiality; 3) the physician should ask the patient if he/she accepts the interpreter; 4) the physician should speak directly to the patient; and 5) the physician should conduct a pre-session discussion with the interpreter. A score of 0 was given when no element of good practice was provided. Examples include “Conduct a physical exam”, “Read the patient's file”, “Ask the patient why he sought asylum in Switzerland”, “Often one can't do better than that”, “learn Swahili”.

The reliability of the coding process was tested in 40 randomly selected records, which were coded independently by two coders (VK and NJP). Kappa statistics were computed to assess agreement. Kappa captures how much agreement there is between raters beyond agreement due to chance: kappa = 0 means that observed agreement can be entirely ascribed to chance, kappa = 0.5 means that observed agreement is mid-way between chance agreement and perfect agreement, and kappa = 1 means perfect agreement. A kappa in the range 0.6 to 0.8 is considered to be “substantial”, and >0.8 “almost perfect.” [22] The raters could agree or disagree on the presence of each of the 5 codes. Each of the 5 codes was distributed into a 2 by 2 table, and a code-specific kappa was obtained (Table 1). The mean kappa was 0.89.

**Table 1:** Code-specific kappas for interpreter vignette (N = 40), and frequencies of respondents (N = 567) who gave each answer.

Codes	kappa	N (%)
Remind interpreter of importance of confidentiality	1	80 (14.1)
Speak directly to the patient	0.95	180 (31.7)
Ask patient if he/she accepts the interpreter	0.88	98 (17.3)
Introduce the interpreter to the patient	0.85	267 (47.1)
Meet with interpreter beforehand to discuss the consultation	0.84	91 (16.0)
No appropriate response	0.80	125 (22.0)

We examined the distribution of the number of good practices mentioned, and compared mean values across respondent characteristics, including self-perceived competence in working with an interpreter. We used analysis of variance to compare groups, and tests for linear trend for ordinal variables.

We considered a p value<0.05 as statistically significant. The analysis was performed with SPSS version 17.

## Results

### Respondent characteristics

We had an overall response rate of 42.7% (619 out of 1450). Response rate was lower among private doctors (29.8%) than among hospital doctors (52.2%) or among medical students (54.2%, p<0.001).

Nearly half of respondents were hospital-based doctors (49.4%); 28.4% were private doctors and 22.1% were medical students. Of the 463 respondents who reported a medical specialty (medical students did not), 35.4% were in general internal or general medicine, 21.0% in psychiatry, 13.6% in medical subspecialties, 7.7% in surgery, 6.3% in gynecology/obstetrics, 5.8% in anesthesiology and 10.2% other. 45.6% of respondents were women, and a majority of respondents (86.6%) were of Swiss nationality. On average, about 30% of respondents' patients were immigrants, defined for the study as patients who were born and raised in a country other than Switzerland (standard deviation 20%, quartiles 15%-30%-40%).

### Respondents frequently encounter language barriers, but are unaware of the problems associated with ad hoc interpreters

More than half of respondents (378, 60.8%) provided one or more answers to the following open-ended question: “What categories of immigrant patients do you have the most difficulty communicating with and understanding?” The most frequently mentioned category was “patients who do not speak French” (n = 128, 34%). Other answers included patients from specific countries, regions or religions (between 4–25%), patients in difficult social/economic situations (6%); and immigrants with specific health problems (2%).

With regards to the list of 15 potential sources of difficulty, insufficient knowledge of the local language (French) by the immigrant patient was the number 1 cause of difficulty: 60.6% of respondents gave it a score of 4 or 5 (on a scale of 1–5). 57.5% placed it among the top 5 causes of difficulty (Table 2). This perception was strongest among the younger respondents: it was rated among the top-5 causes of difficulty by 66.9% of students, 55.8% of hospital doctors, and 51.6% of doctors in private practice (p = 0.038); other characteristics were not related to this variable. Other frequent difficulties included unfocussed complaints by immigrant patients (54.9%), immigrants' illness beliefs that are opposed to medical knowledge (40.7%), insufficient time (40.5%) and immigrant patients' unrealistic expectations (38.7%). Only 32.1% of respondents thought that using the patient's friends or family members as interpreters was a frequent source of difficulty.

**Table 2:** Percent distributions of items identified as sources of difficulties encountered with immigrant patients.

	Rare cause of difficulties			Very frequent cause of difficulties		Top 5 (%)
	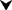				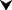	
1) Insufficient knowledge of the local language (French) by the migrant patient	2.5	12.2	24.8	35.0	25.6	57.5
2) Unfocussed complaints by the migrant patient	4.9	14.1	25.6	34.3	21.0	54.9
3) The migrant patient's beliefs are opposed to medical knowledge	13.7	18.3	27.8	27.0	13.3	40.7
4) Insufficient duration of consultations	10.3	19.4	26.0	26.5	17.9	40.5
5) Unrealistic expectations of the migrant patient	11.1	19.6	25.4	31.4	12.5	38.7
6) Doctor's lack of experience with health problems of migrant patients	4.9	18.6	31.9	32.7	11.8	36.6
7) Lack of documents for patients that are translated into languages spoken by migrant patients	6.6	16.2	29.8	32.7	14.7	34.2
8) Doctor's lack of competency to communicate with patients of other languages or cultures	9.4	21.4	27.0	30.1	12.0	31.4
9) Use of friends or family members as interpreters	18.8	24.5	24.7	21.9	10.2	26.3
10) Migrant patient lacks knowledge about how the local health care system functions	9.6	22.9	30.3	27.8	9.4	25.9
11) Doctor's lack of adequate knowledge about the country of origin and culture of migrant patients	7.5	19.4	36.4	29.0	7.8	25.3
12) Doctor's lack of interest of motivation to care for migrant patients	21.7	21.5	23.2	22.2	11.4	23.3
13) Doctor's biases of prejudice against migrant patients	16.4	22.9	27.1	24.7	8.9	21.9
14) Low level of education of the migrant patient	10.6	30.5	31.4	20.6	6.9	18.2
15) Poor adequacy of the Swiss health care system for the needs and expectations of migrant patients	18.3	34.5	32.3	10.7	4.2	11.1

### Respondents consider themselves to be highly competent at working with an interpreter

As previously reported [20], 58.6% of respondents considered themselves to be highly competent (scores of 4 or 5) at working with a professional interpreter, ranking it 5th out of the 14 tasks. This favorable self-assessment was less frequent among students (36.8%) than among hospital doctors (68.6%) and doctors in private practice (58.1%, p<0.001). For comparison, higher overall self-competency ratings were obtained for performing a physical examination (score 4 or 5: 85.0%), obtaining a medical history (78.9%), obtaining a psychosocial history (60.5%), and announcing bad news to a patient (59.0%).

### Few respondents provided examples of good practice working with an interpreter

Of the 619 respondents, 567 (91.6%) gave at least one answer to the interpreter scenario (of the 52 who did not, 25 skipped all 5 scenarios, and 27 skipped the interpreter scenario but answered at least one of the others). Of the 567 respondents, 22% (n = 125) provided no interpreter-related good practices in response to the vignette (score of 0). Thirty-nine percent (n = 223) gave 1 good practice, 30% (n = 169) gave 2 good practices; 8% (n = 44) gave 3 good practices and 1% (n = 6) gave 4 good practices. No respondents mentioned more than 4 good practices in response to the vignette. The most frequently noted good practices were introducing the interpreter to the patient, and speaking directly to the patient (Table 1). The mean number of responses was 1.3 per respondent; it was higher for women than for men (1.4 vs. 1.2, p = 0.02), higher for students than for hospital doctors and doctors in private practice (respectively 1.4, 1.3 and 1.1, p = 0.014), and higher for those who reported more interest in working with immigrants (across the 5 categories of increasing interest: 0.9, 1.0, 1.2, 1.4, and 1.4, p for linear trend 0.002).

The mean number of vignette answers did not vary with self-assessed competency (across the 5 categories of increasing competency: 1.6, 1.2, 1.3, 1.2, and 1.2, p for linear trend 0.26).

Respondents who considered themselves to be “not at all competent” in working with an interpreter (score of 1 on the self-rated scale) gave on average 1.6 valid answers on the vignette, those who rated themselves at 2 gave 1.2 valid answers, those who rated themselves at 3 gave 1.3 valid answers, those who rated themselves at 4 gave 1.2 valid answers, and those who considered themselves to be “perfectly competent (self-rating of 5) gave 1.2 valid answers. The linear trend in the mean number of ratings was not statistically significant (p = 0.26).

## Discussion

Language barriers were a common source of difficulty for our respondents, but there was little acknowledgement of the difficulties inherent in using patients' friends and family members as interpreters. Respondents considered themselves highly competent to work with an interpreter, but few could identify the basics of good practice and there was no association between self-rated competency and knowledge of how to work with an interpreter.

These results may be explained by the fact that ad hoc interpreters (patients' friends and family members or bilingual hospital staff) are used more frequently than professional interpreters and that very few physicians at our hospital have received training in why and how to work with professional interpreters [23], [24]. For many health care professionals, communicating through an interpreter may be seen as an intuitive activity rather than an acquired professional skill with clinical consequences. Lack of awareness of the dangers associated with ad hoc interpreter use and lack of knowledge of the basic elements of effective communication through an interpreter may lead to a false sense of confidence. In fact, several studies suggest that higher levels of confidence in intercultural situations may actually reflect lower insight and awareness [25], [26], [27], [28].

Our method has several potential weaknesses. We used only one vignette to tap into respondents' knowledge of how to work effectively with an interpreter. The vignette required respondents to critique only the beginning of a consultation, and we did not specify that we wanted them to list good practices related to working with an interpreter. The use of different vignettes might have evoked different or additional responses from respondents. We encouraged respondents to provide as many answers as they could, but it may be that those who provided more answers are simply those who were more diligent or had more time to complete the survey. The results might have been different had we pre-determined the number of answers we sought and communicated this to respondents.

And finally, neither vignettes nor self-assessments are good measures of actual skills [29], which can be more accurately assessed using objective measures such as Objective Structured Clinical Examinations (OSCEs), standardized patients, and simulations [30], [31]. However, such objective measures are costly and labor-intensive, and thus more suited to small-scale studies and training contexts. Despite their well-known weaknesses, self-assessments remain a practical evaluation method for large-scale studies. Integrating vignettes such as the one we have tested into surveys may be one way to strengthen self-assessments [32]. Nonetheless, further work is needed to develop and test vignettes which accurately assess respondents' knowledge of good practice.

Finally, our study is limited by a relatively low response rate, so we cannot assume that our results are representative of the local physician population. Nonetheless, we are encouraged by the finding that students scored higher than either hospital or private doctors on knowledge of good practice. This suggests that formal courses on why and how to work with professional interpreters that have been added to the medical curriculum in recent years are having an impact. However, it remains to be seen whether this knowledge will translate into better practice. A recent, unpublished survey indicates that while increasing numbers of physicians know about the interpreter service at our hospital and have received training in how and why to work with professional interpreters, reported practices have changed little between 2006 and 2011 [33]. It is well known that time constraints, habit and institutional barriers also have an important influence on practices [34], [35], [36], [37].

Doctors and medical students in our study named few elements that characterize a well-conducted consultation involving an interpreter, yet most considered themselves to be competent at this activity. A lack of association between self-assessed competency and knowledge of good practice suggests that respondents may lack awareness of what is at stake in interpreted consultations. Training efforts need to challenge the assumption that working with an interpreter is intuitive. Recognition of potential problems and pitfalls of interpreted consultations and awareness of the complex nature medical interpreting may help doctors to adopt the view that working with an interpreter is an important acquired clinical skill. Evaluation of doctors' ability to work effectively with an interpreter should not be limited to self-ratings, but should also include more objective measures of their knowledge and skills.
